# Oculomotor Control in Preterm Infants: Insights from Eye-Tracking Technology

**DOI:** 10.3390/jcm14217742

**Published:** 2025-10-31

**Authors:** María Romero-Sanz, Teresa Pérez-Roche, Marina Vilella Cenis, Adrián Alejandre Escriche, Eduardo Esteban-Ibañez, Marta Ortin Obon, Marta Lacort-Beltrán, Esther Prieto Calvo, Olimpia Castillo Castejón, Victoria Pueyo Royo

**Affiliations:** 1 Ophthalmology Department, Miguel Servet University Hospital, 50009 Zaragoza, Spain; eprietocal@gmail.com (E.P.C.); olimpiacastillocastejon@hotmail.com (O.C.C.); 2 Aragon Institute for Health Research (IIS Aragon), 50009 Zaragoza, Spain; marina.vilella@dive-medical.com (M.V.C.); eduardo.esteban@dive-medical.com (E.E.-I.); marta.lacort@dive-medical.com (M.L.-B.); 3 Barbastro Hospital, 22300 Huesca, Spain; teyeroche@hotmail.com; 4 DIVE Medical S.L., 20014 San Sebastián, Spain; adrian.alejandre@dive-medical.com (A.A.E.); ortin.marta@dive-medical.com (M.O.O.); 5 Department of Microbiology, Pediatrics, Radiology, and Public Health, Faculty of Medicine, University of Zaragoza, 50009 Zaragoza, Spain

**Keywords:** oculomotor control, preterm birth, eye tracking, fixation stability

## Abstract

**Background/Objectives:** This study aims to investigate the development of oculomotor behavior in children born preterm using a DIVE device (Device for an Integral Visual Examination) equipped with eye-tracking technology. Visual and visuo-cognitive measures obtained through eye-tracking technology provide quantitative and sensitive indicators of early neural development, as visual function is one of the earliest cerebral processes to develop postnatally. Design: This is a cross-sectional study. Participants: The study included 428 children aged 0.5 to 14 years. Of these, 214 were born preterm (78 late preterm, born at 32–36 weeks’ gestation, and 136 early preterm, born at less than 32 weeks’ gestation) and 214 were full-term controls, matched by age and gender. **Methods:** All participants underwent a comprehensive ophthalmological assessment. Oculomotor behavior was analyzed using a DIVE device, focusing on fixation duration, saccadic performance and fixation stability. Fixation stability was quantified by the bivariate contour ellipse area for short tasks (BCEA), which measures (in deg^2^) the area of the ellipse enclosing a specified percentage of fixation positions—smaller BCEA values indicate greater fixation stability. We performed a cluster analysis on these oculomotor metrics to identify distinct oculomotor control patterns. **Results:** Preterm children exhibited significantly poorer fixation stability than controls (mean BCEA 0.21 vs. 0.09 logdeg^2^, *p* = 0.004), alongside shorter fixation durations and longer saccadic reaction times. Early preterm children showed more unstable fixations compared to late-preterm and control groups (0.26 vs. 0.12 and 0.09 logdeg^2^, respectively, *p* = 0.001). Cluster analysis revealed three distinct OMC (oculomotor control) patterns: “good,” “impulsive,” and “poor.” Children classified in the “good OMC” cluster demonstrated stable fixations and appropriate saccadic reaction times. In contrast, those in the “impulsive OMC” and “poor OMC” clusters exhibited more unstable fixations and abnormal saccadic times, with the “poor” cluster being significantly associated with extreme prematurity, lower birth weights, and a higher incidence of intraventricular hemorrhage (IVH). **Conclusions:** Preterm birth is associated with measurable oculomotor deficits, most pronounced in early preterm infants, affecting their fixation and saccadic abilities. The findings emphasize the need for targeted interventions to support the development of preterm children, particularly those with “poor” oculomotor control behavior.

## 1. Introduction

Children born preterm could face a broad spectrum of neurodevelopmental deficits that include cognitive, sensory and motor abilities. These challenges include cognitive impairments such as difficulties in attention or executive functions. Moreover, sensory challenges, such as visual deficits, along with motor coordination issues, have an impact on their daily life [1,2,3].

Advances in neonatal care have improved survival rates, but concerns remain about later disabilities in cognition, perception and motor control [4,5].

These limitations are often subtle early in life and may appear later in childhood when it comes to social interaction, focusing attention or learning. Early detection could be essential for stimulating neuroplasticity and avoiding potential complications later in childhood, like social interaction or learning issues [6].

Research indicates that many disabilities in preterm children may be linked to visual abilities, highlighting the need for further investigation into vision as an early-developing cerebral function [7,8,9].

One key requirement for achieving good visual function is the ability to maintain gaze on a target. This cognitive ability, which emerges shortly after birth, helps children understand their environment, including objects and spatial relationships, social communication and motor control. Thus, visual fixation is essential for a child’s motor, social and cognitive performance. Its development occurs in the first months of life, concurrent with the maturation of the fovea and the central nervous system [10,11].

Effective fixation behavior is characterized by central, steady and maintained gaze, involving physiological small eye movements such as micro-saccades, ocular micro-tremor and ocular drifts to prevent visual fading. Maintaining steady fixation involves various mechanisms, including the vestibulo-ocular reflex, optokinetic and smooth pursuit tracking, fusional vergences and accommodation.

In newborns, eye movements are initially purely saccadic, enabling rapid redirection of gaze toward visual stimuli. As early as the second month of life, infants begin to develop smooth pursuit capabilities, and by four months, their horizontal tracking performance approaches that of adults [12].

According to Kaul et al., early visual tracking ability has been linked to later outcomes in cognitive, language, and motor domains [12]. If a child has worse fixation skills than other children of the same age, they could be at risk of neurodevelopmental impairment and thus, at risk of cognitive, perception, and coordination disabilities. Children with learning disorders usually suffer from oculomotor control abnormalities compared to healthy children [13].

Preterm children could have delayed or impaired development of visual tracking. Key neonatal risk factors include lower gestational age, severity of retinopathy of prematurity, bronchopulmonary dysplasia, periventricular leukomalacia and severe brain damage [14]. However, there is not much evidence focused on gaze behavior in children born preterm.

Visual tracking devices could be valuable tools for assessing visual and cognitive skills, including visual attention, gaze stability, saccadic performance, preference vision and memory.

In our study, we use a DIVE (Device for an Integral Visual Examination), with eye-tracking technology, to describe fixational performance in preterm children with different risk factors. Our goal is, therefore, to study the development of fixation behavior in children born preterm. Understanding the main oculomotor development in children born preterm may contribute to an early identification of children at risk of neurocognitive impairment and to minimize its consequences on other learning or behavioral skills.

## 2. Materials and Methods

### 2.1. Participants

This cross-sectional study included a cohort of preterm children and a control group of age- and gender- paired healthy full-term children.

The former were visited in the Pediatric Ophthalmology Department following our preterm children’s vision protocol, while the controls were invited to participate during a visit as part of a vision screening program.

The inclusion criteria for the preterm group were birth before 37 weeks of gestation and chronological age between 6 months and 14 years at the time of the assessment. The control group included children born after 37 weeks of gestation, within the same age range, and without known ocular or neurological disorders, aside from low-grade ametropia. Exclusion criteria for both groups included previous ocular surgery; presence of major congenital malformations, cerebral palsy, or other severe neurological conditions that prevented completion of the eye-tracking evaluation due to lack of cooperation, technical issues, or significant oculomotor abnormalities.

Informed consent was obtained from all parents or legal guardians in accordance with the Declaration of Helsinki. The study protocol was approved by the local ethics committee (CEICA).

Demographic, perinatal, and clinical data were extracted from medical records, including gestational age, birth weight, delivery mode, gender, and the presence of complications such as chorioamnionitis or intraventricular hemorrhage.

### 2.2. Examination

#### 2.2.1. Ophthalmological Assessment

All participants underwent a comprehensive ophthalmological assessment. Visual acuity was assessed monocularly and binocularly in cooperative participants using age-appropriate LEA symbols or optotypes. For infants under 24 months of age and other non-cooperative children, acuity was obtained using the preferential looking test (LEA paddles). The assessment also included an evaluation of ocular motility, refraction under cycloplegia and fundoscopy.

#### 2.2.2. Fixation Stability Assessment

Fixational performance was assessed with the non-invasive DIVE device, which requires only that the child look at images on a screen—no verbal instructions are needed, rather than those provided to the child by the device. The procedure was performed in a quiet room under mesopic illumination. Children sat 50 cm from the screen, either independently or on a parent’s lap if under 3 years old, and they were told to look at different objects on the screen. No eyeglasses were used, and head movements of the children were not restricted to avoid interfering with results.

#### 2.2.3. Equipment

The used device was a DIVE (DIVE Medical S.L., San Sebastián, Spain), with a 12-inch screen, corresponding to a visual angle of 32.56 degrees horizontally and 22.77 degrees vertically.

All the eye movements were recorded during the test by an eye tracker, with a maximum temporal resolution of 120 Hz, which creates by infrared light a vector between the center of the pupil and the corneal reflections to calculate gaze direction.

#### 2.2.4. Calibration and Study

The fixation study, including the previous calibration procedure, lasted less than 5 min in all cases.

Prior to data collection, a calibration procedure of the eye tracker was always performed. Each child was asked to fixate on a cartoon image of an animal with an associated sound, which appeared at 9 different locations across the screen, one at a time, with no overlap between them. The calibration procedure was repeated until at least six valid calibration points were obtained, and the overall calibration quality reached at least 3 out of 5 points.

After calibration, fixation tasks were carried out. The fixation target was a stylized image of an animal. Eight different visual stimuli were displayed in each sequence, as a combination of features with binomial categories: size (0.91 degrees and 1.95 degrees), sound (with or without buzzing sound), and motion (static or with slight movement simulating a vibration).

Each sequence began with a central fixation target, and after 3 s, 8 peripheral stimuli were randomly displayed throughout the screen with a fixed distance of 9.26 degrees every two consecutive stimuli. Each stimulus was presented for 3 s, with no stimulus overlap.

We performed the test binocularly, but data from each eye were registered separately. Only data from the right eye were analyzed in this study.

#### 2.2.5. Analysis of Fixations

For analysis, fixations were identified using a well-established dispersion-based algorithm. Fixation stability was calculated by the bivariate contour ellipse area (BCEA), quantifying the area in squared degrees (deg^2^) of the ellipse containing a certain percentage of the fixation points. We report the logarithmic transformation of the BCEA (logdeg2), so a smaller value for BCEA is indicative of greater fixation stability.

BCEA = 2 × k × π × σ_x_ × σ_y_ × (1 − p^2^)^1/2^, where σ_x_ is the standard deviation of the horizontal eye position, σ_y_ is the standard deviation of the vertical eye position, p is the Pearson product-moment correlation coefficient of the horizontal and vertical eye positions, and k is obtained from P as P = 1 − e^−k^.

Fixational performance was described by fixation stability during the 2 central seconds of the trial, mean fixation duration, and saccadic reaction time, defined as the time between the stimulus appearance and the onset of saccadic movement towards the stimulus.

#### 2.2.6. Statistical Analysis

All data were analyzed using SPSS 26.0 statistical software (SPSS Inc., Chicago, IL, USA).

The data were tested for normal distribution using the Kolmogorov–Smirnov test. Due to the normal distribution of the sample, parametric tests were used.

Descriptive and visual outcomes were described by their mean and standard deviation. Quantitative measurements were compared among groups using Student’s *t*-test and analysis of variance (ANOVA).

Cluster analysis was employed as a methodology to categorize participants based on oculomotor control variables. Cluster analysis is a statistical technique used to identify groups or clusters within a dataset by assessing the similarities between observations.

The optimal number of clusters was determined by using the Bayesian Information Criterion (BIC) and Akaike Information Criterion (AIC), which indicated that three clusters were the most appropriate representation. In addition to statistical criteria, we searched for clinical interpretation of the identified clusters. In this context, three different clusters were identified according to the k-means.

Demographic and perinatal characteristics were compared across clusters using ANOVA or Chi-square tests. Additionally, odds ratios were calculated to assess the association between specific risk factors and oculomotor profiles identified in the clustering analysis.

## 3. Results

Two cohorts of 214 children (114 males and 100 females) were finally included in the study, with overall good cooperation.

Age at testing did not differ between the two study groups.

The cohort of preterm children had a mean birth weight of 1496.14 g and a mean gestational age of 30.54 weeks, while in the control group, the mean birth weight was 3231.94 g and the mean gestational age was 39.14 weeks.

Most children achieved age-appropriate visual acuity. When assessed with the ETDRS test, visual acuity was significantly better in term children compared to preterm children in both eyes—right eye (0.79 ± 0.25 vs. 0.70 ± 0.31; *p* = 0.049) and left eye (0.81 ± 0.24 vs. 0.65 ± 0.30; *p* = 0.003). By contrast, no group differences were observed with age-appropriate LEA symbols (*p* = 0.825/0.943) or LEA grating tests (*p* = 0.683/0.914).

In contrast, refractive errors were significantly more frequent among preterm children. The mean spherical equivalent was lower in the preterm group for both eyes (OD: 1.17 D vs. 1.57 D, *p* = 0.032; OS: 1.18 D vs. 1.64 D, *p* = 0.009). Astigmatism was also higher in preterm children (OD: 1.04 D vs. 0.68 D, *p* < 0.001; OS: 1.09 D vs. 0.65 D, *p* < 0.001).

These characteristics are shown in Table 1.

Gaze stability was poorer in children born preterm. They presented more unstable fixations, shorter median fixation duration, although not statistically significant, and longer saccadic reaction times. The results are summarized in Table 2.

We divided the preterm cohort into two groups according to their gestational age at birth: late preterm (32–37 weeks of gestational age; *n* = 78) and early preterm (<32 gestational age; *n* = 136). 

Although late preterm children are more prevalent, in our study, we have studied more early preterm children because, as part of our clinical protocol, we monitor visual development throughout childhood in early preterm children, but only in high-risk late preterm children.

As shown in Table 3, when analyzed by subgroups, early preterm children (with a mean gestational age of 28.48 weeks) showed more unstable fixations than late preterm (34.66 weeks) and control groups (39.24 weeks). However, no differences were found in the median duration of fixations or saccadic performance.

Since preterm children in our study suffered from more ocular comorbidities, impaired oculomotor control might be related to coexisting disorders, such as refractive errors or strabismus.

**Figure 1:**
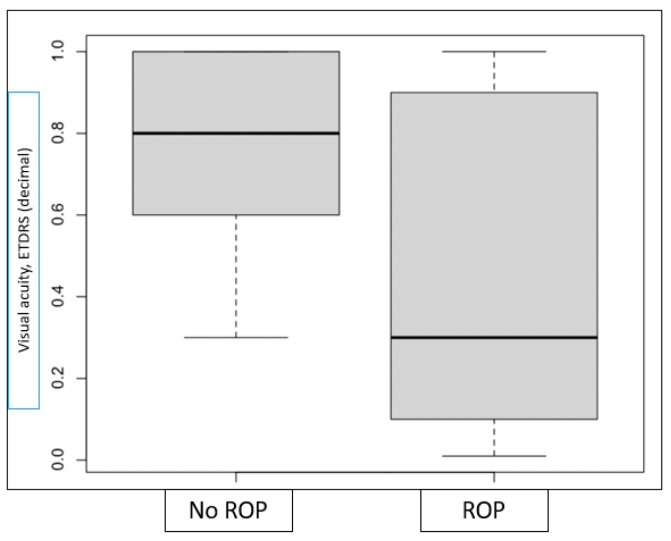
Differences in visual acuity measured with the ETDRS optotype between children with and without a history of ROP in the neonatal period.

Of the total number of children born with a GA below 32 weeks (136 children) and therefore at risk of developing ROP, 20 presented the condition. In all cases, it was treated with diode laser photocoagulation in both eyes, with a favorable outcome. Children with a history of ROP had a mean GA at birth of 26.1 weeks (SD 2.00 weeks), compared to those without ROP, who had a mean GA of 28.72 weeks (SD 1.90 weeks), and a birth weight of 838.25 g (SD 242.58 g) versus 1225.51 g (SD 337.05 g). Although all visual parameters were worse in children with a history of ROP than in those without, the differences only reached statistical significance for VA measured using the ETDRS optotype (Figure 1). The small sample size in both groups likely prevented the detection of additional between-group differences.

To quantify the impact of prematurity without any other source of bias, a second analysis was performed, including only children with normal visual development (no refractive errors or strabismus and no other visual disorder) and their matched controls.

Seventy-four preterm children were excluded due to ocular comorbidities. Despite removing the potential effect of any visual disorder on oculomotor performance, preterm children with a normal full ophthalmological exam performed worse than their controls. Gaze stability was poorer in children born preterm. Also, shorter median fixation time and longer saccadic reaction time were detected. Results are shown in Table 4.

**Figure 2:**
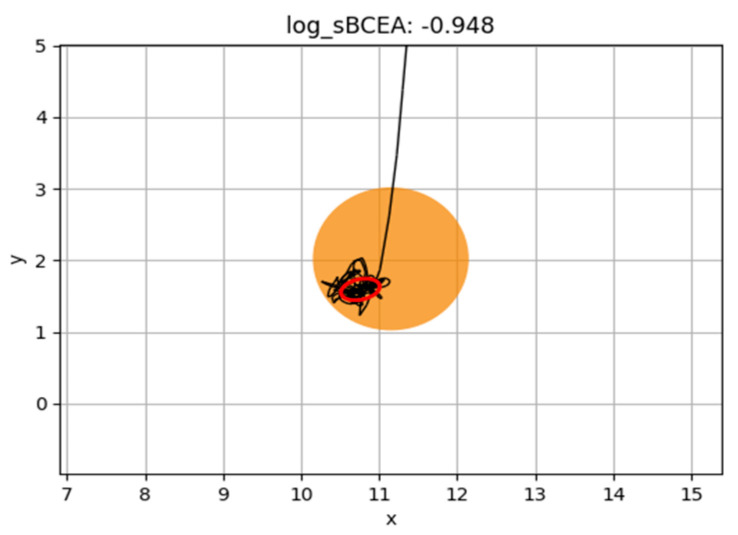
Representation of BCEA of a child with good fixation. We represent in red color BCEA and in black color all the other ocular movements (fixations, saccades, non-classified eye movements…).

**Figure 3:**
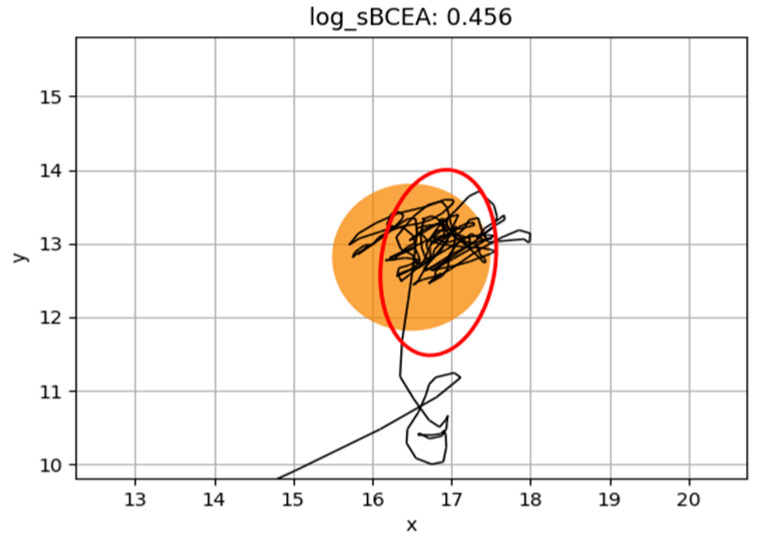
Representation of BCEA of a child with poor fixation. We represent in red color BCEA and in black color all the other ocular movements (fixations, saccades, non-classified eye movements…).

As illustrated in Figure 2 and Figure 3, children with better fixation stability show a smaller BCEA area, with saccades confined within this region, reflecting a more accurate and efficient oculomotor control. In contrast, children with poor fixation present a larger BCEA area and more scattered eye movements, consistent with reduced precision and stability of fixation.

Based on their fixational and saccadic performance, three clusters were identified, representing different oculomotor phenotypes: “poor”, “impulsive” and “good” oculomotor performance.

**Figure 4:**
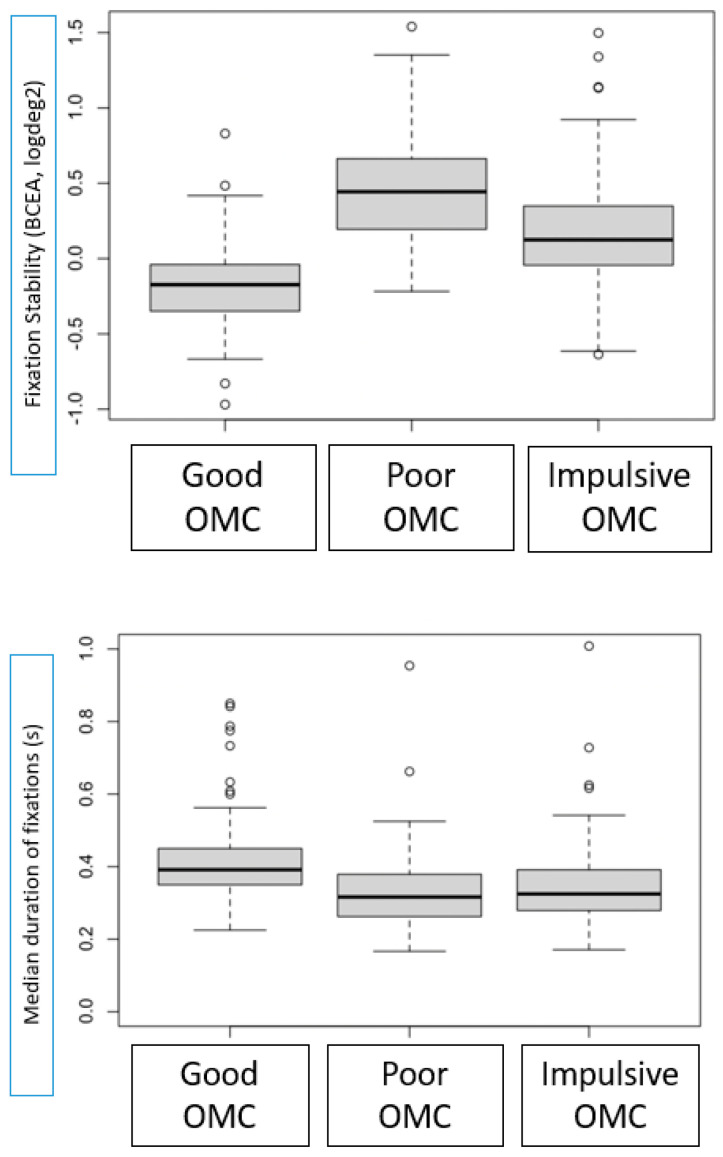

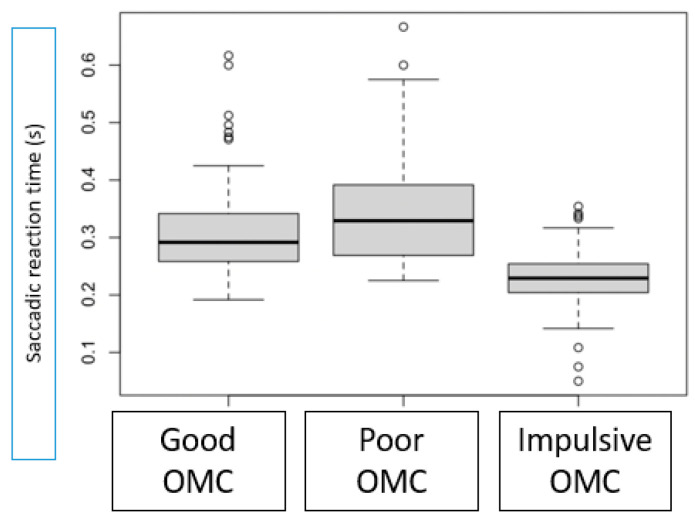
Boxplots of oculomotor performance across the three clusters of oculomotor control (OMC). OMC 1: Good, OMC 2: Poor, OMC 3: Impulsive. The graphs display fixation stability (BCEA, logDeg^2^), median duration of fixation (ms), and saccadic reaction times (ms). Lower BCEA values indicate better fixation stability, whereas longer fixation duration and shorter saccadic latencies reflect more efficient oculomotor control.

As shown in Figure 4, children with “good oculomotor performance” exhibited stable, prolonged fixations and appropriate saccadic reaction times, while those with “impulsive oculomotor performance” had unstable, short fixations and abnormally brief saccadic reaction times. The “poor oculomotor performance” group demonstrated the shortest, most unstable fixations and increased saccadic reaction times. In Table 5, we present oculomotor control outcomes from the three identified oculomotor control phenotypes: cluster 1 or “good oculomotor performance” (*n* = 140), cluster 2 or “impulsive oculomotor performance” (*n* = 140) and cluster 3 or “poor oculomotor performance” (*n* = 137).

We present demographic characteristics of the three oculomotor control clusters. As shown in Table 6, children with poor oculomotor control behavior tend to present lower gestational age at birth, lower birth weight and increased rate of intraventricular hemorrhage. According to our data, the presence of intraventricular hemorrhage during the neonatal stage had the highest impact on oculomotor control later in childhood (OR 2.5 (95% CI 1.2–5.4)).

## 4. Discussion

### 4.1. Main Finding

Premature birth places the child in a situation of vulnerability against potential neurodevelopmental disorders. Among many other cognitive, motor or sensory deficits, oculomotor control is often compromised, leading to difficulties maintaining fixation on static targets, tracking moving objects or coordinating eye movements with other neurological functions. Such impairments can not only affect their visual function but also have an impact on their cognitive development, learning skills or social interactions.

Few studies have analyzed fixational quality in preterm children. The literature suggests that preterm birth can have a large impact on visual processing functions and brain development [3]. Abilities such as tracking visual objects, maintaining a steady gaze, and quickly shifting attention between targets develop rapidly during the first six months of life and play a crucial role in the development of cognitive functions like attention, memory, and problem-solving. Healthy former preterm children generally are slightly delayed in the development of visual motion processing compared to term-born controls. As a result, preterm children are at increased risk of cognitive disabilities [3,15,16].

The observed difference in visual acuity between preterm and term-born children was only evident when measured with the ETDRS test, but not with LEA symbols or LEA grating paddles. This discrepancy can be explained by the different sensitivity and cognitive demands of these visual acuity assessments.

The ETDRS chart provides a quantitative, high-resolution evaluation of letter recognition acuity and requires greater visual discrimination, attention, and cognitive processing, which are more demanding tasks for older children.

In contrast, LEA symbols and grating paddles are developmentally adapted tests for younger or less cooperative children. They rely on symbol recognition or preferential looking, providing a less sensitive measure of visual resolution.

The absence of group differences in LEA-based tests but their presence in ETDRS assessments may reflect that higher-order visual processing and fine discrimination become more relevant, and more vulnerable, in older preterm children, once tasks require sustained fixation, selective attention, and cortical integration.

### 4.2. Importance of Fixation Stability

Visual fixation is a skill that is essential for the child’s motor, social and cognitive performance. The clear image of the objects projected in the retina when fixation is stable, centered in the macula, allows children to interact with the environment and learn.

To hold moving images in the fovea, two types of movements are needed: saccades and smooth pursuit eye movements. Saccades are fast and brief movements, short-lasting that quickly shift new images into the fovea. Smooth pursuit eye movements are slow and continuous to track any motion of the visual target. The mechanisms involved in the control of these movements involve several cortical and subcortical brain regions, including the superior colliculus, cerebellum and reticular formation [17].

Gaze tracking is achieved by a combination of head and eye movements. Therefore, it is important not to restrict head movement during the assessment, as this could interfere with gaze tracking.

Disruptions in these processes, as observed in children with poor oculomotor control, can lead to significant challenges not only in visual tasks but also in broader cognitive functions.

### 4.3. Development of Visual Fixation Physiology

Visual fixation is not a passive process but rather an active and dynamic function that depends on the integrity and interaction of multiple central nervous system structures. It involves two main pathways: the thalamic-cortical pathway and the subcortical stream.

The thalamic-cortical pathway begins with cones and rods in the retina detecting an image and encoding it into neural signals that travel along the optic nerve via the lateral geniculate nucleus to the primary visual cortex in the occipital lobe. From there, two distinct streams go to the associative visual cortex: the ventral stream, which processes “what” information like faces, shapes, and colors in the temporal lobe; and the dorsal stream, which processes “where” and “how” information like motion and spatial relations in the parietal lobe [6,18].

Nowadays, ocular fixation has been a subject of interest. It is a dynamic process; the eyes are not completely still during fixation. Continuous bustle movements, such as ocular drift and micro-saccades, are detectable. The prefrontal cortex plays an active role in cancelling unwanted saccades and controlling micro-saccades. Eye position during fixation is actively controlled and depends on bilateral activity in the superior colliculi and cerebellum; disruptions in these circuits can cause systematic deviations in eye position during both fixation and smooth pursuit eye movements. The vestibulo-ocular and optokinetic reflexes are involved to stabilize the image in the retina [17].

Recent evidence suggests that early extrauterine visual experience contributes positively to the maturation of visual fixation. In low-risk preterm infants, early exposure to visual stimuli after birth accelerates the development of ocular motor skills like fixation and tracking, compared to term infants evaluated at 48 h of life [19,20].

This supports the notion that visual experience plays a key role in shaping early oculomotor behavior. However, while some components (like basic tracking or fixation) appear to benefit from this early stimulation, more complex, cortically mediated aspects—such as attention at distance—still show a lag compared to term-born counterparts [19]. Therefore, visual fixation is not only anatomically driven but also experience-dependent during early life.

### 4.4. Consequences of Impaired Visual Fixation

Stable fixation is essential for the accurate intake and processing of visual information, and its impairment can have cascading effects on higher cognitive functions. Unstable visual fixation and defective saccadic movements can be found in certain neurologic diseases. Neurodevelopmental impairment can be caused by any process that damages the central nervous system. Preterm children, especially those born before 32 weeks, are particularly vulnerable due to the immaturity of cortical and subcortical circuits at birth. Early disruptions such as hypoxic–ischemic insults, periventricular leukomalacia, and intraventricular hemorrhage are frequent in this population and have been associated with cerebral visual impairment (CVI) and poor oculomotor control [21].

Damage to the prefrontal cortex may cause difficulties with the suppression of saccades, resulting in poor control with more unstable fixations and too short saccadic reaction times [22]. Cerebellar lesions have been shown to cause saccadic dysmetria [23] and reduced smooth pursuit velocity at the end of the open-loop period [24].

But the cognitive shift seen in preterm newborns is not only the result of an anatomical (brain injuries such as venous infarctions, detectable using magnetic resonance imaging) but also a functional (cortical network disruption at the visual pathways microstructural level) problem. Probably both underlying mechanisms are involved, but the last studies underline that despite the absence of visible brain damage evaluated with conventional magnetic resonance imaging, a delay in visual processing may be present [25].

Eye-tracking studies have identified early deficits in attention orientation and fixation control in preterm infants. At 12 months, very preterm infants display a reduced ability to sustain attention on visual targets and exhibit delayed processing speed compared to term peers, even when they score within the average range on standardized developmental scales [18].

These deficits are thought to reflect early dysfunction in the neural networks supporting attentional control and oculomotor regulation [12]. Notably, even in the absence of major structural brain abnormalities detectable on MRI, alterations at the microstructural or functional level may result in significant delays in visual processing [21].

Thus, the early assessment of visual fixation and attentional behavior through objective tools like eye-tracking could serve as a sensitive marker of neurodevelopmental vulnerability. Moreover, these findings reinforce the potential utility of early visual stimulation and targeted intervention in high-risk populations to improve oculomotor outcomes and, potentially, cognitive development trajectories.

### 4.5. Different Oculomotor Control Patterns

Our research, using advanced clustering techniques based on BIC and AIC criteria, identified three different patterns of oculomotor control performance: “good”, “impulsive” and “poor”.

By applying cluster analysis to categorize children according to their oculomotor performance, we gained a clearer understanding of the variability in visual processing among preterm children. This approach enabled the identification of specific subgroups with similar oculomotor profiles, offering valuable insight for developing more individualized and targeted interventions.

Children in the “good” cluster exhibited stable fixations and accurate saccadic reaction times. In contrast, those in the “impulsive” and “poor” clusters demonstrated greater instability in fixation and abnormal saccadic latencies. Notably, the “poor” cluster was associated with extreme prematurity, lower birth weight, and a higher incidence of intraventricular hemorrhage, factors that had a marked negative impact on oculomotor performance.

These results emphasize the importance of early, tailored interventions for children at greater risk, particularly those born preterm or with neonatal complications, to promote better oculomotor functioning and overall developmental outcomes.

This is especially relevant considering that a substantial portion of early learning depends on vision. Difficulties in maintaining fixation or efficiently shifting gaze toward new stimuli can impair a child’s ability to engage with their environment and acquire new skills. Early detection and intervention for oculomotor impairments could therefore improve not only visual function but also cognitive and social development.

### 4.6. Future Directions

Our study aims to assess the consequences of prematurity in oculomotor control, contributing to the growing body of evidence highlighting the importance of early visual processing skills in overall neurodevelopment, particularly in children born preterm.

However, more research is needed to fully understand the long-term consequences of impaired oculomotor control and to develop effective interventions. Future studies could explore how early oculomotor control interventions influence other aspects of neurodevelopment, such as language acquisition, executive function, and social skills.

Further research studies are required to assess whether these oculomotor control patterns may be related to any neurodevelopmental issues, such as attention deficit or hyperactivity.

By addressing these gaps in knowledge, we can better support the developmental needs of preterm children and improve their long-term outcomes.

## 5. Conclusions

Visual and visual processing screening and monitoring throughout childhood ought to be encouraged in children born prematurely, not only to decrease the deleterious consequences of prematurity, but also to achieve optimal development.

Our study highlights the importance of understanding and addressing specific deficits in oculomotor control among preterm infants. By identifying different oculomotor control profiles, we can tailor interventions better to meet individual needs. For instance, children in the “poor” oculomotor control group may benefit from intensive, specialized therapies to improve fixation stability and saccadic control. Similarly, those in the “impulsive” group might require interventions to enhance impulse control and visual processing.

Implementing such personalized strategies is crucial for supporting the developmental trajectory of preterm children.

## Figures and Tables

**Table 1:** Demographic and visual characteristics of both study groups and the significance of the comparisons. Abbreviations: D, diopters. Values are expressed as mean ± standard deviation.

	Preterm Children	Full-Term Children	
Mean birth weight (g)	1496.14 ± 613.19	3231.94 ± 407.94	*p* < 0.001
Mean gestational age at birth (weeks)	30.54 ± 3.5	39.14 ± 1.15	*p* < 0.001
Adjusted age at testing (years)	4.17 ± 3.07	4.06 ± 3.08	*p* = 0.897
Spherical Equivalent right eye (D)	1.57 ± 2.38	1.17 ± 1.24	*p* < 0.001
Spherical Equivalent left eye (D)	1.64 ± 2.24	1.18 ± 1.21	*p* < 0.001
Astigmatism right eye (D)	1.04 ± 0.91	0.68 ± 0.43	*p* < 0.001
Astigmatism left eye (D)	1.09 ± 0.94	0.65 ± 0.42	*p* < 0.001

**Table 2:** Comparison of oculomotor control outcomes in preterm and term-born children. Abbreviations: BCEA, standardized bivariate contour ellipse area; log deg^2^, logarithm of squared degrees; s, seconds. Values are expressed as mean ± standard deviation.

	Fixation Stability (BCEA, log deg^2^)	Median Fixation Duration (s)	Saccadic Reaction Time (s)
Preterm	0.21 ± 0.4	0.35 ± 0.1	0.30 ± 0.09
Control	0.09 ± 0.4	0.37 ± 0.1	0.28 ± 0.08
	*p* = 0.004	*p* = 0.057	*p* = 0.032

**Table 3:** Comparison of oculomotor control outcomes in early preterm vs. late preterm vs. control children. Abbreviations: BCEA, standardized bivariate contour ellipse area; log deg^2^, logarithm of squared degrees; s, seconds. Values are expressed as mean ± standard deviation.

	Mean Gestational Age (Weeks)	Fixation Stability (BCEA, log deg^2^)	Median Fixation Duration (s)	Saccadic Reaction Time (s)
Early preterm	28.48	0.26 ± 0.4	0.33 ± 0.09	0.29 ± 0.09
Late preterm	34.66	0.12 ± 0.4	0.35 ± 0.13	0.30 ±0.08
Control	39.24	0.09 ± 0.4	0.37 ± 0.12	0.28 ± 0.08
		*p* = 0.001	*p* = 0.132	*p* = 0.098

**Table 4:** Oculomotor control outcomes in children with normal ophthalmological examination comparing preterm and full-term children. Abbreviations: BCEA, standardized bivariate contour ellipse area; log deg^2^, logarithm of squared degrees. Values are expressed as mean ± standard deviation.

	Fixation Stability (BCEA, log deg^2^)	Median Fixation Duration (s)	Saccadic Reaction Time (s)
Preterm	0.22 ± 0.39	0.34 ± 0.09	0.30 ± 0.08
Control	0.07 ± 0.39	0.37 ± 0.11	0.28 ± 0.08
	*p* = 0.001	*p* = 0.021	*p* = 0.031

**Table 5:** Oculomotor control outcomes from the three identified oculomotor control phenotypes. Abbreviations: OMC, oculomotor control; BCEA, standardized bivariate contour ellipse area; log deg^2^, logarithm of squared degrees; ms, milliseconds. Values are expressed as mean ± standard deviation.

	Cluster 1 “Poor OMC”	Cluster 2 “Impulsive OMC”	Cluster 3 “Good OMC”	*p*-Value
Fixation stability, BCEA (logdeg^2^)	0.45 ± 0.36	0.18 ± 0.36	−0.17 ± 0.27	<0.001
Fixation duration (ms)	0.31 ± 0.07	0.32 ± 0.06	0.46 ± 0.13	<0.001
Saccadic reaction time (ms)	0.34 ± 0.09	0.23 ± 0.05	0.31 ± 0.08	<0.001
*N*	140	140	137	

**Table 6:** Demographic characteristics of the three oculomotor control clusters. Values are expressed as mean ± standard deviation or percentages, as appropriate. Abbreviations: OMC, oculomotor control.

	Cluster 1 “Poor OMC”	Cluster 2 “Impulsive OMC”	Cluster 3 “Good OMC”	*p*-Value
Age (y)	4.37 ± 3.33	4.07 ± 2.81	3.81 ± 3.03	0.314
Gender (male/female)	61:79	67:70	68:72	0.221
Gestational age (weeks)	33.89 ± 5.18	35.20 ± 5.07	35.47 ± 4.75	0.019
Birth weight (g)	2159.23 ± 1018.62	2446.29 ± 1052.96	2490.97 ± 940.73	0.012
Prematurity (rate)	61.4%	43.8%	44.3%	0.004
Extreme prematurity (rate)	39.3%	29.9%	26.4%	0.016
Maternal corioamnionitis	20.9%	16.7%	16.1%	0.704
Intraventricular haemorrhage (rate)	15.7%	13.9%	6.4%	0.041
Bronchopulmonary dysplasia	23.2%	25%	12.9%	0.190
Visual disorders (refraction, strabismus)	23.6%	10.2%	12.1%	0.004
*N*	140	140	137	

## Data Availability

The raw data supporting the conclusions of this article will be made available by the authors on request.
